# The Promise of Aggregation-Induced Emission Luminogens for Detecting COVID-19

**DOI:** 10.3389/fimmu.2021.635558

**Published:** 2021-02-16

**Authors:** Zongwei Liu, Ting Meng, Xiaofang Tang, Ran Tian, Weijiang Guan

**Affiliations:** ^1^Department of Respiratory Medicine, Lianyungang Hospital of Traditional Chinese Medicine, Affiliated Hospital of Nanjing University of Chinese Medicine, Lianyungang, China; ^2^The First Clinical Medical College, Nanjing University of Chinese Medicine, Nanjing, China; ^3^State Key Laboratory of Chemical Resource Engineering, College of Chemistry, Beijing University of Chemical Technology, Beijing, China; ^4^Public Laboratory, Tianjin Medical University Cancer Institute and Hospital, National Clinical Research Center for Cancer, Key Laboratory of Cancer Prevention and Therapy, Tianjin's Clinical Research Center for Cancer, Tianjin, China

**Keywords:** COVID-19, diagnosis, aggregation-induced emission, immunoassay, nucleic acid

## Abstract

The long-term pandemic of coronavirus disease 2019 (COVID-19) requires sensitive and accurate diagnostic assays to detect severe acute respiratory syndrome coronavirus 2 (SARS-CoV-2) virus and SARS-CoV-2 antibodies in infected individuals. Currently, RNA of SARS-CoV-2 virus is mainly detected by reverse transcription-polymerase chain reaction (RT-PCR)-based nucleic acid assays, while SARS-CoV-2 antigen and antibody are identified by immunological assays. Both nucleic acid assays and immunological assays rely on the luminescence signals of specific luminescence probes for qualitative and quantitative detection. The exploration of novel luminescence probes will play a crucial role in improving the detection sensitivity of the assays. As innate probes, aggregation-induced emission (AIE) luminogens (AIEgens) exhibit negligible luminescence in the free state but enhanced luminescence in the aggregated or restricted states. Moreover, AIEgen-based nanoparticles (AIE dots) offer efficient luminescence, good biocompatibility and water solubility, and superior photostability. Both AIEgens and AIE dots have been widely used for high-performance detection of biomolecules and small molecules, chemical/biological imaging, and medical therapeutics. In this review, the availability of AIEgens and AIE dots in nucleic acid assays and immunological assays are enumerated and discussed. By building a bridge between AIE materials and COVID-19, we hope to inspire researchers to use AIE materials as a powerful weapon against COVID-19.

## Introduction

Coronavirus disease 2019 (COVID-19), caused by severe acute respiratory syndrome coronavirus 2 (SARS-CoV-2) virus, has spread over 216 countries/regions and resulted in more than 84 million infected cases and nearly 1.8 million deaths (1). SARS-CoV-2 is an RNA virus and has five open reading frames (ORFs) to encode the hemagglutinin esterase dimer protein (HE), the spike protein (S), the envelope protein (E), the glycosylated membrane protein (M), and the nucleocapsid protein (N), respectively (2, 3). The pathogenesis of SARS-CoV-2 infection contains two courses: virus entry cell and virus replicates. During the cellular entry of the virus, the S protein plays a key role through host recognition and is closely associated with the human receptor angiotensin-converting enzyme 2 (ACE2), which is highly expressed in the lung, stomach, small intestine, colon, kidney, and lymph nodes (4–6). The human immune system responds to viral infection *via* the innate and adaptive immune systems, gradually producing antibodies against SARS-CoV-2 (7). Despite the use of multiple therapies over nearly a year, there are still no effective treatments for SARS-CoV-2 infection. Thus, the most effective and economical strategy is to identify infected individuals and prevent healthy individuals from coming into contact with infected individuals.

Current detection methods can be divided into two categories: nucleic acid assays and immunological assays (8–12). Nucleic acid assays directly detect the presence of SARS-CoV-2 virus in the upper respiratory tract during the first days of infection. Among nucleic acid assays, reverse transcription-polymerase chain reaction (RT-PCR)-based assays are currently considered the gold standard and are generally composed of six steps: (1) collection of respiratory samples using swabs, (2) inactivation of SARS-CoV-2 using lysis buffer, (3) purification of the SARS-CoV-2 RNA, (4) conversion of the purified RNA to complementary DNA (cDNA) using reverse transcriptase, (5) amplification of the specific regions of cDNA using primers, and (6) detection of the amplified cDNA using luminescence probes (11). The design of primers and probes targeting different regions of the SARS-CoV-2 genome can significantly affect the detection sensitivity, as low-performance nucleic acid amplifications and probes may generate false negatives. Moreover, nucleic acid assays require specialized instruments and complicated operations and are of little help in identifying past infections and monitoring infection status and immune progress (13). On the other hand, immunological assays with simpler design enable convenient and rapid detection of SARS-CoV-2 antigen, immunoglobulin M (IgM) and immunoglobulin G (IgG) antibodies in the serum of infected individuals (8). IgM and IgG are generally produced after 5–10 d upon SARS-CoV-2 infection and last for several weeks (8). Immunological assays rely on the formation of immune complexes between the antibody and antigen. In the presence of appropriate substrates, luminescence probes labeled on the antibody or antigen can be activated to produce a given luminescence that is capable of both qualitative observation under a UV lamp and qualitative analysis on a spectrophotometer. Obviously, the detection sensitivity of nucleic acid assays and immunological assays is closely related to the performance of the luminescence probes. However, conventional luminescence probes inevitably encounter the aggregation-caused quenching (ACQ) problem at high target concentrations, greatly limiting their performance.

Aggregation-induced emission (AIE), first proposed by Tang in 2001, is a diametrically opposite phenomenon to ACQ (14). The luminogen with AIE characteristics is named AIEgen, which has no/low luminescence in the molecularly dispersed state but enhanced luminescence in the aggregated state (14–18). Further mechanistic studies indicate that strong luminescence can be also achieved by restricting the intramolecular motions (RIM) of the AIEgens in the molecularly dispersed state (16–18). After 20 years of outstanding development, a great family of AIEgens has been established, ranging from twisted conjugated AIEgens to planar conjugated AIEgens and irregular non-conjugated AIEgens (14–21). Moreover, AIEgen-based nanoparticles (AIE dots) have been well-developed to obtain highly efficient luminescence (22–25). Both AIEgens and AIE dots are widely used for the high-performance detection of small molecules and biomolecules, chemical/biological imaging, and medical therapeutics (15, 22–33). Undoubtedly, some of them can be used for nucleic acid assays and immunological assays. Therefore, this review aims to bridge the gap between AIE materials and COVID-19 detection and proposes AIE materials as potential diagnostic weapons against COVID-19. Possible candidates for nucleic acid assays and immunological assays are summarized. This knowledge may contribute to the development of advanced diagnostic kits that are effective for more sensitive assays.

## AIEgens Exhibit Great Potential For Nucleic Acid Assays

Since the number of viruses reach the maximum in the first few days of infection, nucleic acid assays can provide the earliest information to determine the presence or absence of the SARS-CoV-2 virus (9). Although the protocols and advances of nucleic acid assays have been reviewed (8–12), little attention is paid to the design of luminescence probes for highly sensitive detection of the amplified cDNA. Typically, commercial double-stranded DNA (dsDNA) binding dyes (e.g., SYBR Green) show low luminescence in the free state but enhanced luminescence upon insertion into the dsDNA sequence (9). Their luminescence intensity increases quantitatively with the exponential amplification of cDNA. However, the short Stokes shift (<30 nm) allows a large overlap between the absorption and emission spectra, which leads to self-absorption or inner-filter effects, thus reducing the signal-to-noise ratio and the detection sensitivity (30). There is no doubt that further innovations in producing more efficient luminescence signals and outputs could significantly improve the detection sensitivity. Therefore, this section focuses on the great potential of using AIEgen-based DNA probes to improve the sensitivity of current nucleic acid assays.

**Figure 1:**
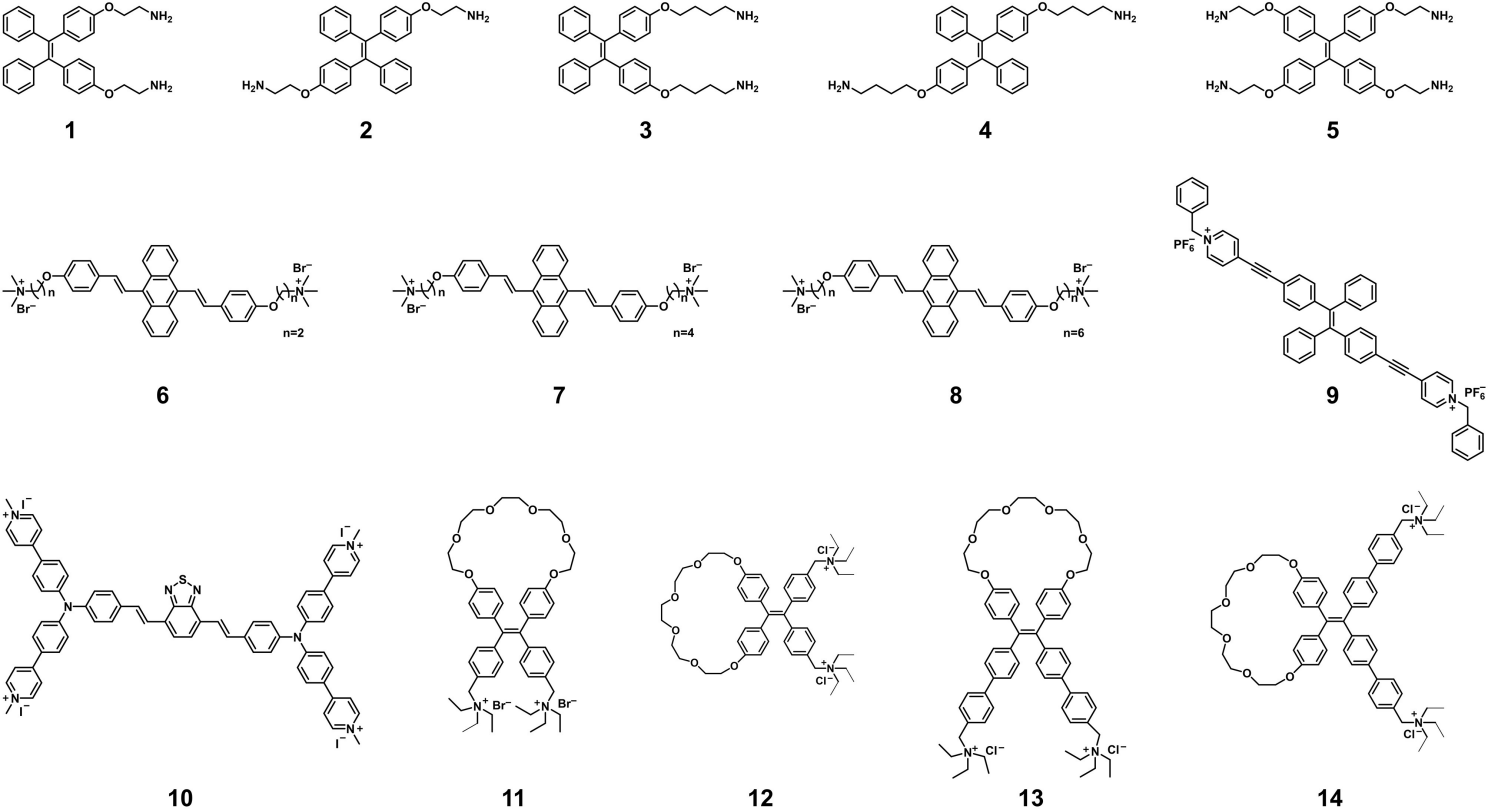
Chemical structures of AIEgens for the detection of nucleic acids. Adapted and modified with permission from Xu et al. (39) (Copyright 2014 American Chemical Society), Wang et al. (44) (Copyright 2016 Wiley-VCH Verlag GmbH & Co. KGaA, Weinheim), Wang et al. (47) (Copyright 2018 Royal Society of Chemistry), Gao et al. (48) (Copyright 2019 American Chemical Society), and Yuan et al. (52) (Copyright 2020 American Chemical Society).

Similar to SYBR Green, AIEgen-based DNA probes show low luminescence in the free state but enhanced luminescence upon binding to dsDNA, which is caused by RIM (32–51). AIEgen-based DNA probes (Figure 1) consist of two parts: AIE-active groups for producing light-up signals and targeting groups for binding to DNA (34–53). The AIE-active groups have a larger Stokes shift (>100 nm) compared to SYBR Green, which prevents the reabsorption or inner-filter effects as well as aggregation-caused quenching, thereby improving sensitivity. The effect of AIEgen structures on DNA detection has been systematically revealed by studying the number of binding groups, the length of hydrophobic linking groups and the molecular conformation. Taking the AIEgens **1**, **3**, and **5** as examples (39, 42), their cationic amino-groups can endow them with good water solubility to avoid the aggregation and fluorescence of the AIE-active tetraphenylethene (TPE) groups. Meanwhile, these amino-modified AIEgens can firmly bind negatively charged dsDNA through electrostatic interactions and hydrogen bonds, resulting in TPE aggregation to display strong fluorescence. The detection limit of AIEgen **1** and AIEgen **3** with two amino-groups were much lower than that of AIEgen **5** with four amino-groups, indicating that the extra two amino-groups cannot make AIEgens bind dsDNA more firmly but reduce the aggregation of TPE groups. On the other hand, AIEgen **3** with longer hydrophobic linking groups showed higher sensitivity than AIEgen **1** with shorter hydrophobic linking groups. This result demonstrated that stronger hydrophobic interactions between AIEgens can facilitate the aggregation of TPE groups, leading to the enhanced fluorescence and higher sensitivity. Based on the revealed relationship between the AIEgen structures and detection sensitivity, AIEgens **11–14** with restriction of double-bond rotation were developed to further improve the sensitivity of sensing DNA (52). In addition, the conformational change of AIEgen **10** results in a two-color fluorescent signal from red to green, enabling selective and sensitive determination of dsDNA (48). When bound to anionic macromolecules, AIEgen **10** emits a bright red fluorescence at about 640 nm. Interestingly, dsDNA can wrap around AIEgen **10** and change its molecular conformation, resulting in a change in conjugation and bright green fluorescence of about 537 nm. From these published studies, it is understood that AIEgens with two binding sites, proper hydrophobicity and variable conformation enable sensitive detection of dsDNA. To facilitate the utilization of AIEgen-based DNA probes, their detection limits for dsDNA are summarized in Table 1. It is clear that AIEgen-based DNA probes are like a vast treasure trove, and some of them can serve as reliable and sensitive fluorescence probes that can be quickly adapted to SARS-CoV-2 PCR assays after proper calibration. At the same time, more efforts are needed to develop new AIEgens for other nucleic acid-based SARS-CoV-2 assays.

**Table 1:** Summary of detection limits for dsDNA.

**AIEgens**	**Target analyte**	**LOD**	**References**
1	75–300 bp	4 ng/band	(39)
	50 bp	10.5 ng/band	
	35 bp	15 ng/band	
5	50 bp	>42 ng/band	
3	75–300 bp	1 ng/band	
	50 bp	<3.5 ng/band	
	35 bp	5 ng/band	
	25 bp	7.5 ng/band	
	20 bp	8 ng/band	
	15 bp	19 ng/band	
	10 bp	<24 ng/band	
1	75–300 bp	1 ng/band	(42)
	50 bp	<3.5 ng/band	
	35 bp	2.5 ng/band	
	25 bp	5 ng/band	
	20 bp	5.3 ng/band	
	15 bp	9.5 ng/band	
	10 bp	12 ng/band	
6 + GO1	T1	0.17 × 10^−9^ M	(44)
6 + GO2		11.8 × 10^−9^ M	
6 + GO3		15.5 × 10^−9^ M	
7 + GO1		2.7 × 10^−9^ M	
7 + GO2		22.9 × 10^−9^ M	
7 + GO3		26.1 × 10^−9^ M	
8 + GO1		2.1 × 10^−9^ M	
8 + GO2		24.1 × 10^−9^ M	
8 + GO3		22.4 × 10^−9^ M	
9	DNA (cas: 9007-49-2)	0.02 μg/mL	(47)
10	ct DNA	1.75 ng/mL	(48)
	WT HIV DNA	7.2 × 10^−11^ M	
11	FS-DNA	123 pM	(52)
13		74 pM	
12		496 pM	
14		235 pM	
11	ct DNA	7.3 ng/mL	
13		1.6 ng/mL	
12		22 ng/mL	
14		2.1 ng/mL	

## AIEgens Exhibit Great Potential For Immunological Assays

Theoretically, if the luminescence signals can increase with the concentrations of luminescent labels without the ACQ problem, the detection sensitivity for immunological assays will be continuously improved. This is exactly the strength of AIE, therefore, this section focuses on the great potential of using AIEgens and AIE dots to improve the sensitivity of current immunoassays, such as enzyme-linked immunosorbent assays (ELISA) and lateral flow immunoassays.

**Figure 2:**
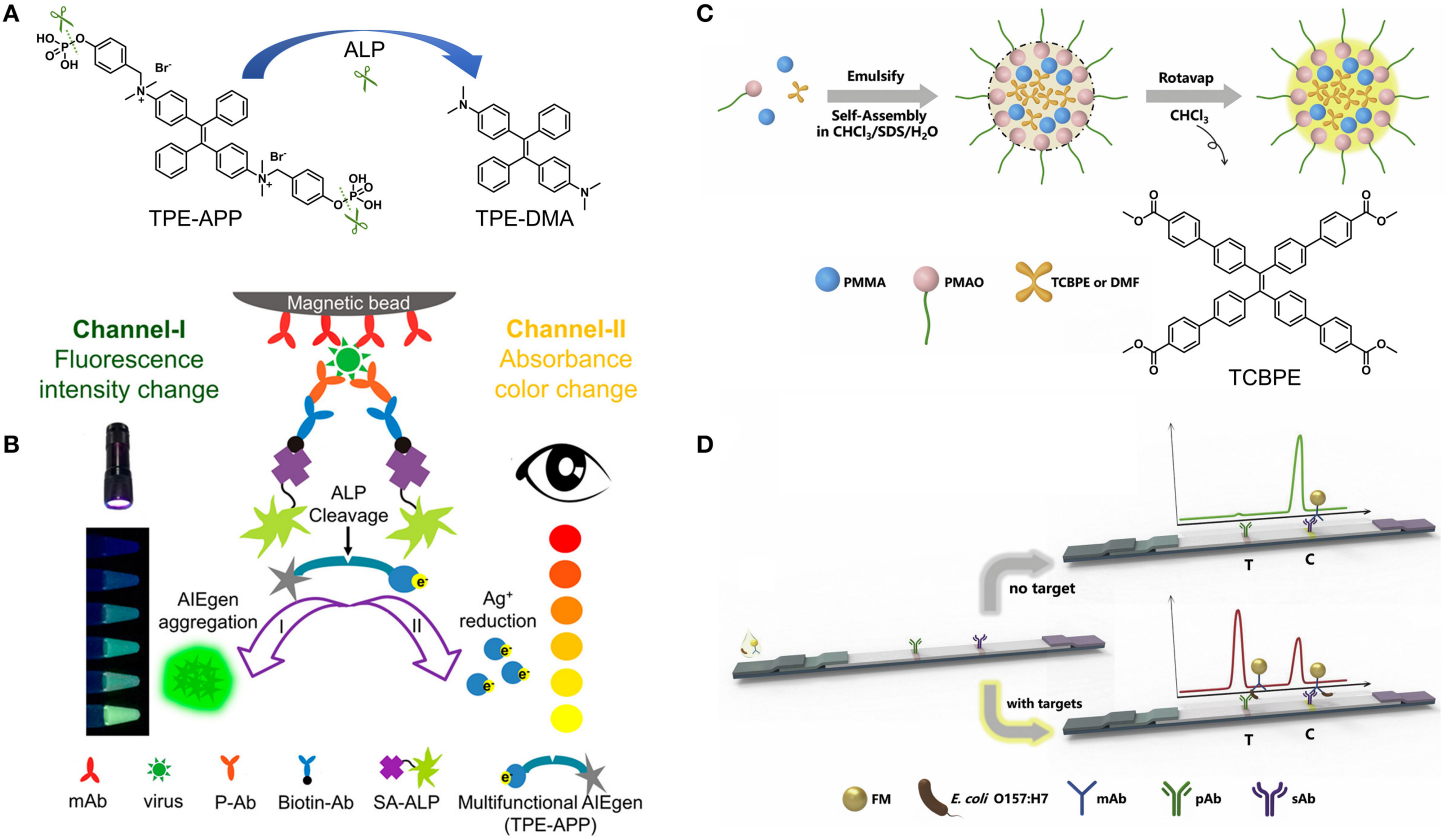
**(A)** ALP-catalyzed hydrolysis of TPE-APP to TPE-DMA. **(B)** Schematic illustration of fluorescent and plasmonic colorimetric dual-modality for virus detection based on the ALP-catalyzed hydrolysis of TPE-APP. Reprinted with permission from Xiong et al. (56). Copyright 2018 American Chemical Society. **(C)** One-pot synthesis strategy and **(D)** the principle of the immunochromatographic assay based on AIEFM. Reprinted with permission from Zhang et al. (59). Copyright 2019 Elsevier B.V.

ELISA is typically performed on the plate wells coated with the SARS-CoV-2 viral protein. The SARS-CoV-2 antibody (if present) can specifically bind the SARS-CoV-2 viral protein to form antibody–protein complex, which can be detected by an additional tracer antibody. This assay is very fast and results can be obtained within 1–5 h. Various AIEgens with switchable luminescence have been successfully used in ELISA (54–58), and can be good candidates for constructing ELISA to detect SARS-CoV-2 infection. For example, a dual-modality readout immunoassay platform was developed using AIEgen-based signal unit consisting of streptavidin-alkaline phosphatase (SA-ALP), TPE-APP with enzyme cleavage sites, and gold nanoparticles (56). When the immunocapture unit composed of magnetic beads, anti-VP1 monoclonal antibodies (mAb-VP1), rabbit polyclonal antibodies (P-Ab) and biotinylated antibodies (Biotin-Ab) captures the target EV71 virus, SA-ALP will promote the hydrolysis of the water-soluble TPE-APP to produce the water-insoluble TPE-DMA, resulting in the aggregation of TPE-DMA in water and the emission of bright fluorescence (Figures 2A,B). The fluorescence turn-on mode gives the immunoassay platform a lower detection limit, as low as 1.4 copies/μL (56). At the same time, the hydrolysis of TPE-APP can mediate the reduction of silver ions, thereby generating silver nanoshells on the surface of gold nanoparticles. The resulting nanoshells cause a significant plasmon color change, which can be clearly recognized by the naked eye in a wide range from 1.3 × 10^3^ to 2.5 × 10^6^ copies/μL (56). Additionally, the immunocapture unit can capture different wanted targets (e.g., H7N9 virus and Zika virus) through conjugating suitable antibodies to achieve accurate and ultrasensitive detection. It can be anticipated that the proposed protocol could inspire and stimulate new developments in clinical diagnosis of SARS-CoV-2 viral protein with high accuracy and sensitivity by varying AIEgens with switchable luminescence. Compared to most turn-off fluorescent probes, the fluorescence turn-on property gives AIEgens a unique advantage in terms of signal reliability and sensitivity.

Lateral flow immunoassay is typically performed in a paper-like membrane strip with two lines (8). The test line is coated with colorimetric or fluorescent material-antibody conjugates, and the control line is coated with capture antibodies. After depositing the test person's blood on the membrane strip, the SARS-CoV-2 antibody (if present) can specifically bind to the colorimetric or fluorescent material-antibody conjugate while flowing through the test line. The resulting complex continues to move until it is immobilized by the capture antibodies on the control line, causing a visible line under daylight or ultraviolet (UV) light. It is worth noting that the sensitivity of the fluorescent signal is much higher than that of the colorimetric signal. By virtue of the AIE feature, AIEgens can be integrated into nanoparticles at high concentrations, resulting in one AIE dot for labeling in response to one binding unit (59–61). For example, AIEgen-based fluorescent microsphere (AIEFM) was obtained by self-assembly of poly(methyl methacrylate) (PMMA), poly (maleic anhydride-alt-1-octadecene) (PMAO), sodium dodecyl sulfate (SDS), and AIE-active TCBPE (Figures 2C,D). The hydrophilic part of PMAO acts outward as a functional group to conjugate with anti-*E. coli* O157:H7 monoclonal antibody, forming the fluorescent immunoprobe (AIEFM-mAb) (59). Anti-*E. coli* O157:H7 polyclonal antibody (pAb) was immobilized on the test line (T) of the nitrocellulose membrane while goat anti-mouse antibody (sAb) was immobilized on the control line (C). The target *E. coli* O157:H7 (if present) can be specifically captured by mixing with AIEFM-mAb to generate bright fluorescence on both the test line and control line. Otherwise, AIEFM-mAb can only be captured by sAb on the control line to emit bright fluorescence. The advantage of using AIE-active TCBPE is that the higher the loading of TCBPE, the stronger the fluorescence signal and the higher the detection sensitivity. As a result, the AIEFM endows the lateral flow immunosensor with a very low detection limit, down to 3.98 × 10^3^ CFU/mL, which is about 10 times lower than that of the two commercial FMs (59). Undoubtedly, the above strategy is also applicable to the construction of lateral flow immunoassay kits for detecting SARS-CoV-2 antigen and antibody. Higher performance detection can be achieved not only by using different AIEgens for AIEFM, but also by using other AIE dots. In addition, some news reported that lateral flow immunoassay kits for SARS-CoV-2 antigen or antibody detection have been developed through the use of highly luminescent AIE dots (62, 63). Therefore, it is reasonable to conclude that AIEgens-/AIE dots-based SARS-CoV-2 immunoassay kits can be designed and produced with higher sensitivity and lower detection limit.

To facilitate the understanding and utilization of AIE dots, current AIE dots are divide into two categories: pure AIE dots and composite AIE dots (22–25). The commonly used preparation method is anti-solvent precipitation, which is achieved by adding an anti-solvent in which AIEgens are poorly soluble or by reducing the volume of a solvent in which AIEgens are well soluble. This method is convenient and principally applicable to any AIEgen to form the pure AIE dots. However, the formed pure AIE dots usually have difficult to control particle morphology (e.g., spheres, ellipsoids, and bulk amorphous), particle size, and surface modification, making them difficult to perform desired and specific applications. To overcome these difficulties, more sophisticated molecular designs and synthetic efforts are required to obtain functional AIEgens. On the other hand, the composite AIE dots are formed from encapsulating various AIEgens into the different amphiphile matrices. The amphiphile matrices provide not only stability for the AIE dots and water solubility for the organic AIEgens, but also enhance the control of morphology and size of AIE dots. By simultaneously precipitating AIEgens and amphiphilic matrices, spherical AIE dots of uniform particle sizes are usually produced, which can be easily further modified. The commercial matrices include, but are not limited to: poly(ethylene glycol) derivatives (e.g., 1,2-distearoyl-sn-glycero-3-phosphoethanolamine-N-[methoxy(polyethylene glycol)-2000] (DSPE-PEG_2000_), poly(ethylene glycol)-*b*-polystyrene (PEG-*b*-PS), 1,2-distearoyl-sn-glycero-3-phosphoethanolamine-N-[folate(polyethylene glycol)-5000] (DSPE-PEG_5000_-folate), etc.), bovine serum albumin (BSA), and biocompatible surfactants (e.g., pluronic F127 and lecithin). It is worth noting that the internal dispersion of AIEgens throughout the amphiphilic matrix may be stochastic and vary from molecular to aggregate distributions when preparing and using the composite AIE dots. Compared to the pure AIE dots, the composite AIE dots preserve the properties of both materials to offer a number of unique advantages: (1) synthetic efforts are minimized by reducing the hydrophilic groups; (2) luminescence performance are well-tuned by mixing various AIEgens in different concentrations; and (3) high specificity and responsiveness are achieved by using the amphiphilic matrices to conjugate with diverse linkers.

## Conclusion

**Scheme 1:**
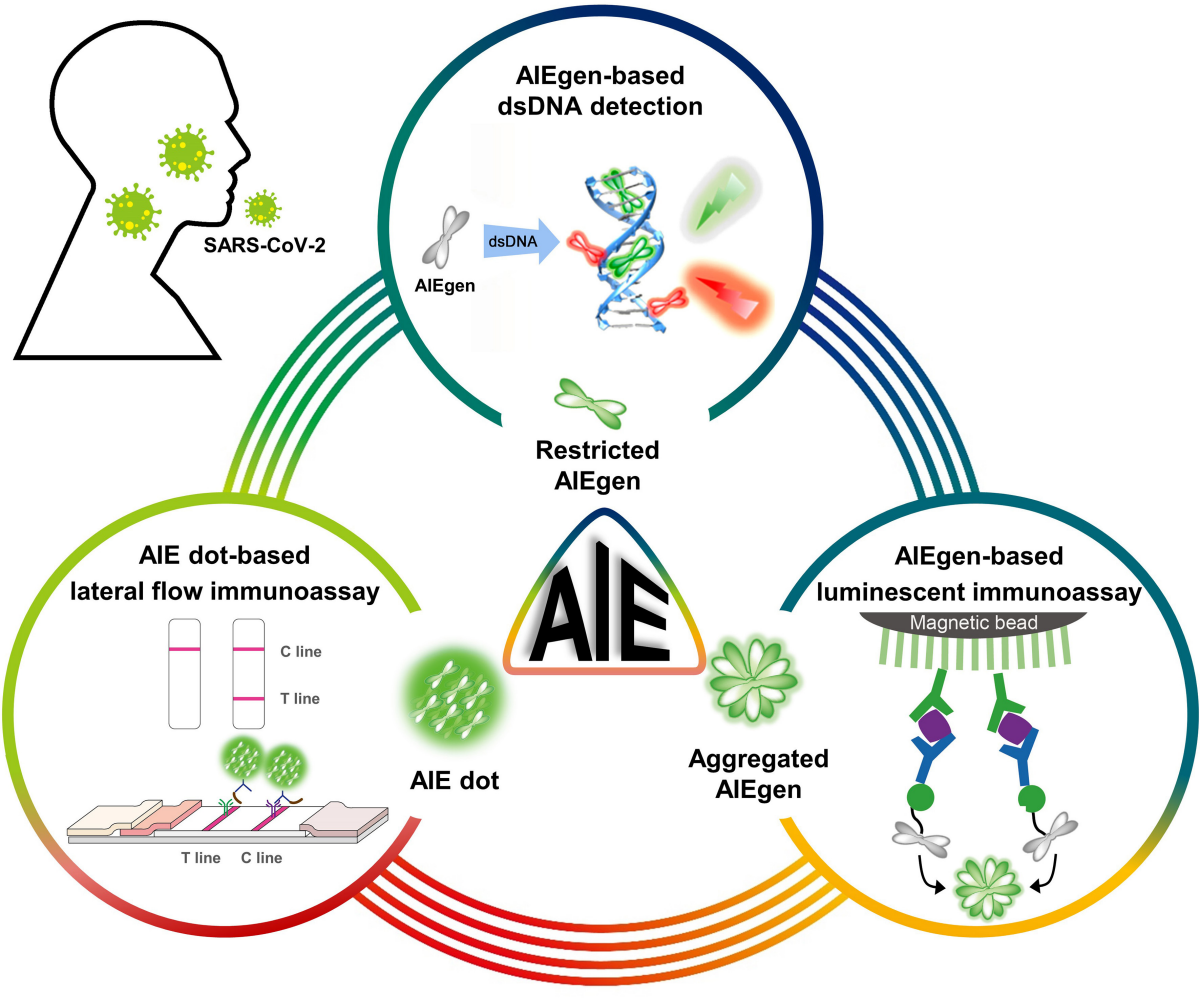
Schematic diagram of AIEgens and AIE dots as potential tools for COVID-19 detection. Partially cited from Gao et al. (48), Xiong et al. (56), and Zhang et al. (59).

Global health is facing the most dangerous situation regarding the COVID-19 pandemic. Owing to the large number of infected cases worldwide, the rapid long-term spread, and lack of effective treatments, there is a continuing need for more sensitive, low-cost, rapid, and point-of-care diagnostic kits to identify cases of SARS-CoV-2 infection in communities and individuals without advanced facilities. To date, while RT-PCR assays have become the primary technique for detection of SARS-CoV-2 RNA, other nucleic acid assays including isothermal amplification assays, hybridization microarray assays, amplicon-based metagenomics sequencing, and the cutting-edge CRISPR-related technologies have also been developed (64). Challenges related to current nucleic acid assays mainly include improving detection sensitivity to eliminate false negatives and performing rapid and point-of-care tests outside of sophisticated laboratories. On the other hand, immunological assays are capable of detecting both SARS-CoV-2 antigens and antibodies. Immunoassays for antigens enable direct, rapid, low-cost, and point-of-care detection of SARS-CoV-2 infection at an early stage. Since antigen proteins are unable to be amplified like viral genes, it is crucial to develop new fluorescent probes to improve sensitivity. Immunoassays for antibodies can provide valuable knowledge about the course and extent of the immune response, which are useful in further identifying infected individuals and their close contacts. In particular, the immune persistence of healthy participants can be well-tracked during the vaccination phase. Based on the challenges present in these assays, this review highlights the inherent advantages of AIEgens and AIE dots that can help improve sensitivity (Scheme 1). AIEgens used in nucleic acid assays show negligible fluorescence in the free state but turn-on fluorescence upon binding to dsDNA, and the fluorescence signals increase with the binding numbers without the ACQ problem. Similarly, the AIE dots used in the immunoassays can continuously increase the fluorescence intensity by increasing the loading concentration of AIEgens.

In addition, there are some issues that need further improvement when AIE materials are used to COVID-19 detection: (1) development of the sequence-specific DNA probes labeled with AIE dots and quenchers; (2) promotion of AIE dots-based lateral flow immunoassay kits for COVID-19 clinical testing; (3) diversification of AIEgens and AIE dots that can be directly labeled with highly targeted molecules (e.g., antibodies); and (4) further attempts to use AIE materials for studying immune mechanisms and pharmacological treatment of COVID-19 infection (65). With these solid foundations and promising possibilities, there is no doubt that AIE materials should be regarded as a promising anti-SARS-CoV-2 weapon.

## Author Contributions

All authors listed have made a substantial, direct and intellectual contribution to the work, and approved it for publication.

## Conflict of Interest

The authors declare that the research was conducted in the absence of any commercial or financial relationships that could be construed as a potential conflict of interest.
